# Prevention of Opioid Misuse and Abuse Through Effective Pain Management in Patients With Chronic Pain: An Umbrella Systematic Review

**DOI:** 10.7759/cureus.80906

**Published:** 2025-03-20

**Authors:** Sana Sultana, Safeera Khan

**Affiliations:** 1 General Practice, California Institute of Behavioral Neurosciences and Psychology, Fairfield, USA; 2 Internal Medicine, California Institute of Behavioral Neurosciences and Psychology, Fairfield, USA

**Keywords:** chronic pain, effective pain management, opioid abuse, opioid misuse, rehabilitation

## Abstract

Chronic pain is a condition that frequently affects patients and communities. There are several treatment options available, including both pharmacological and non-pharmacological. Opioid prescriptions have increased over the past few years, and long-term use of opioids leads to an increased risk of opioid misuse and death due to overdose. This systematic review discusses the effective pain management options in chronic non-cancer pain patients that may help prevent opioid use and misuse. We searched PubMed, PubMed Central (PMC), Medical Literature Analysis and Retrieval System Online (MEDLINE), Multidisciplinary Digital Publishing Institute (MDPI), and Google Scholar for relevant literature. The different results were screened by the application of eligibility criteria, and 15 papers were finalized for review. These papers discussed the different pain management options, physician guidelines, and efforts to reduce opioid misuse, the importance of pill counting, and the involvement of multidisciplinary care teams in pain management. However, most of these papers were reviews over a short duration. The effects of emotions on chronic pain have been discussed along with the multidisciplinary pain rehabilitation treatment options that have improved patients' overall function. The reviewed research demonstrated positive outcomes of spinal cord stimulation in chronic low back pain, thereby reducing opioid use. However, further research is needed to explore more treatment options for chronic pain that can adequately reduce pain and prevent opioid use.

## Introduction and background

The International Association for the Study of Pain (IASP) defines pain as “an emotional experience that is unpleasant and that may be associated with tissue damage” and defines chronic pain as “pain continuing beyond normal healing time for a tissue, which is expected to be three months” [1]. Greater pain severity is correlated with poor patient outcomes, and patients’ physical and mental health is affected by chronic pain [2]. There is a huge burden on individuals and communities due to chronic pain [3].

Chronic pain treatment has several interventions, including nerve blocks, surgeries, implantable drug delivery systems, and nerve stimulators; however, the primary treatment for pain is oral analgesics, which include acetaminophen, non-steroidal anti-inflammatory drugs (NSAIDs), and opioids [4]. The Centers for Disease Control and Prevention (CDC) guideline’s first recommendations are that non-pharmacologic and non-opioid pharmacologic therapy should be tried first and preferred for chronic pain [5]. A variety of pharmacological and non-pharmacological interventions are used in the management of chronic pain; however, most of these treatments have not been assessed in long-term studies, and there is a wide range in patient presentation, course of illness, and response to treatment in chronic pain [6].

Increased opioid prescriptions have been associated with a tremendous rise in fatal opioid overdoses, with a total of more than 16,000 deaths per year (CDC, 2015) [6]. The most extensively used analgesic medications in the management of chronic non-cancer pain (CNCP) are opioids [7]. There is also growing evidence that opioids have only limited effectiveness in the management of CNCP, and the increased availability of prescription opioids has contributed to an increase in opioid addiction cases and overdose deaths [8].

Reviews have found reports of incomplete pain relief from existing treatments in patients with chronic pain, and patients complain of a significant impact on their overall quality of life affecting their general activity, mood, and enjoyment, as well as their ability to sleep, work, and walk [9,10]. Due to the increased presentation of chronic pain in our society, and in order to manage these conditions, safe and effective treatment options are needed [11].

In this systematic review, we aim to explore the options for the prevention of opioid misuse/abuse with effective chronic pain management. We also aim to explore different strategies and pain management options that might be beneficial to reduce long-term opioid use.

## Review

Methods

Our systematic review was conducted according to the Preferred Reporting Items for Systemic Reviews and Meta-Analysis (PRISMA) 2020 guidelines [12].

Search Sources and Strategy

Electronic databases were utilized, including PubMed, PubMed Central (PMC), Medical Literature Analysis and Retrieval System Online (MEDLINE), Multidisciplinary Digital Publishing Institute (MDPI), and Google Scholar for relevant literature searches. The keywords used for search in different combinations were Chronic pain, Pain management, Adverse effects of pain management, and Opioid misuse/abuse. Another strategy was developed for the PubMed MeSH database: (((("Opioid-Related Disorders/complications"[Mesh] OR "Opioid-Related Disorders/drug therapy"[Mesh] OR "Opioid-Related Disorders/rehabilitation"[Mesh] OR "Opioid-Related Disorders/therapy"[Mesh])) AND "Opioid-Related Disorders"[Majr]) AND ("Chronic Pain"[Mesh])) AND (("Pain Management/adverse effects"[Majr] OR "Pain Management/methods"[Majr])), along with the mentioned keywords in PubMed. Table 1 shows the search strategy and the number of identified papers. 

**Table 1:** Search strategies, keywords used, and number of papers identified. MDPI: Multidisciplinary Digital Publishing Institute; MeSH: Medical Subject Headings

Keywords/search strategy	Databases used	Number of results
(((( "Opioid-Related Disorders/complications"[Mesh] OR "Opioid-Related Disorders/drug therapy"[Mesh] OR "Opioid-Related Disorders/rehabilitation"[Mesh] OR "Opioid-Related Disorders/therapy"[Mesh] )) AND "Opioid-Related Disorders"[Majr]) AND ("Chronic Pain"[Mesh])) AND (( "Pain Management/adverse effects"[Majr] OR "Pain Management/methods"[Majr]))	PubMed MeSH database	38
Prevention of opioid misuse/abuse with effective pain management in chronic pain	PubMed	364
Prevention of opioid misuse/abuse with effective, timely outpatient pain management in patients with chronic pain	Google Scholar	516
Opioid misuse/abuse	MDPI	25

Inclusion and Exclusion Criteria

Full-text peer-reviewed articles in English from the past ten years and involving the adult population were included. Grey literature and articles involving the pediatric population were excluded.

Selection Process

All shortlisted articles were transferred to EndNote (Clarivate Analytics, Philadelphia, Pennsylvania, USA), and the duplicates were removed. Two independent authors (SS and SK) performed all the relevant screening and searches per the PRISMA guidelines. Further evaluation of articles was done by reading the full-text papers and applying the inclusion and exclusion criteria. Articles fulfilling the criteria were qualified for the list.

Quality Assessment of the Studies

Quality assessment of qualified articles was done with various quality appraisal tools. All the co-authors of this study did the quality check. The Assessment of Multiple Systematic Review (AMSTAR) tool was used for systematic reviews [13]. In contrast, the narrative reviews were evaluated using the Scale for the Assessment of Narrative Review (SANRA) checklist [14].

Data Collection Process

The first and second authors extracted the data separately with equal involvement of all the other co-authors assessing the primary outcomes and finalizing the data using the data extraction questionnaire.

Results

Study Identification and Selection

**Figure 1:**
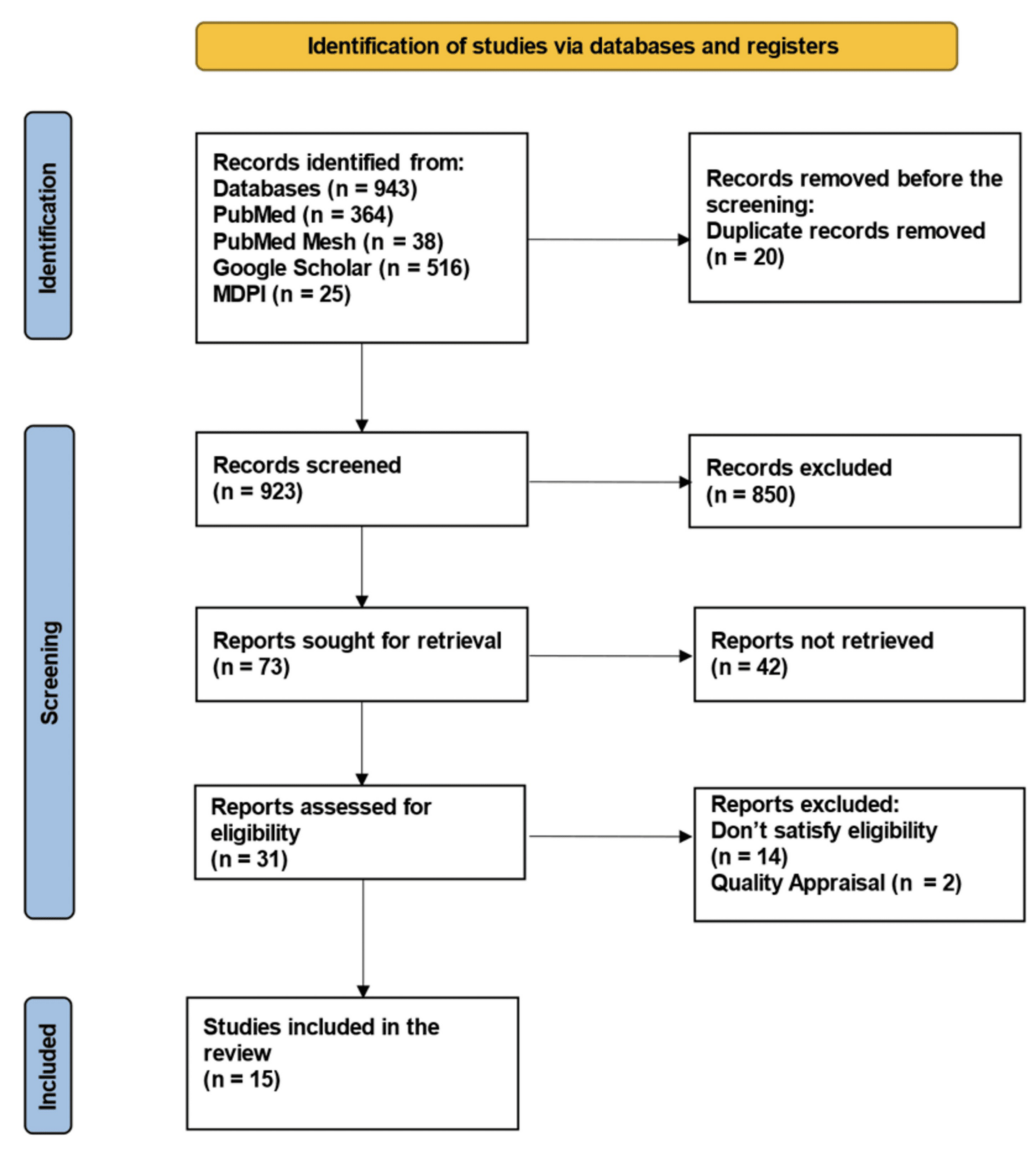
PRISMA flowchart presenting the process of article selection. PRISMA: Preferred Reporting Items for Systematic Reviews and Meta-Analyses; MDPI: Multidisciplinary Digital Publishing Institute; MeSH: Medical Subject Headings

A total of 943 relevant articles were identified using the following databases: PubMed (364), PubMed Mesh (38), Google Scholar (516), and MDPI (25). A total of 20 duplicates were removed before screening them in detail. A total of 31 articles were shortlisted after reviewing the titles and retrieving full texts. The 31 shortlisted articles were assessed for eligibility, and the quality assessment was done by the relevant quality assessment tools, resulting in 15 finalized articles for review. The PRISMA flowchart shows the selection process (Figure 1).

Outcomes Measured

The primary outcomes assessed from the finalized research articles were the possible prevention of opioid use and misuse, with effective pain management in patients diagnosed with chronic pain. The secondary outcomes assessed were the benefits of using alternative pain management options, including the involvement of multidisciplinary pain rehabilitation teams for chronic non-cancer pain patients. The psychological context of chronic pain and the possible benefits of multidisciplinary pain rehabilitation were observed.

Study Characteristics

Fifteen papers were reviewed. Out of these finalized studies, 11 were reviews, three were systematic reviews, and one was a narrative review. All patients involved had chronic non-cancer pain, including patients who had undergone a surgical procedure. One of the studies focused on using spinal cord stimulation in managing chronic low back pain, along with four studies focusing on the psychological context of pain, co-occurring mental health conditions, involvement of physical therapists, and efficacy of pill counting during chronic pain management. One of the studies focused on chronic post-surgical pain, and four focused on the approach to pain, guidelines for physicians on initiating and maintaining opioid therapy, and practitioner training.

Discussion

Chronic pain is a condition that affects physical well-being as well as emotional well-being, has mental impacts on patients, and diminishes their quality of life [11]. Commonly used non-opioid treatments for chronic pain are non-steroidal anti-inflammatory agents (NSAIDs) and acetaminophen, anticonvulsants, antidepressants, topical therapies (topical analgesics like lidocaine), immunomodulators (disease-modifying anti-rheumatic drugs (DMARDs)), muscle relaxants, cannabinoids, nerve blocks, spinal cord stimulation (SCS), and opioids [11]. A combination therapy of acetaminophen or NSAIDs and small amounts of opioids is a good opioid-sparing option. Fixed-dose combinations are available for use [15]. Pill counting also has benefits in treating pain [16].

To reduce opioid use and misuse, professional societies recommend non-pharmacologic treatments when suitable [11]. Movement-based therapies, integrative therapies, behavioral therapies, and procedures are the non-pharmacologic interventions that can be used. The Agency for Healthcare Research and Quality (AHRQ) systematically reviewed non-pharmacologic interventions and found multiple interventions for six common chronic pain conditions [17]. There is some evidence for the short-term effectiveness of non-pharmacologic and non-opioid therapy for chronic pain that also has less overdose risk [5]. Usually, opioid therapy is initiated without a clear treatment plan, and renewal of prescription is done as patients complain of persistent pain, leading to unintentionally prolonged opioid therapy [18]. Table 2 shows the 2017 Canadian Guideline for Opioids for Chronic Non-cancer Pain [19].

**Table 2:** The 2017 Canadian Guideline for Opioids for Chronic Non-cancer Pain. mg: milligram

Scenario	Recommendations
1. Chronic non-cancer pain therapy for patients	Non-opioid pharmacotherapy and non-pharmacological therapy in place of trial of opioids
2. Persistent pain with optimum non-opioid therapy, no substance use disorder, no psychiatric disorder	Adding a trial of opioids suggested
3. Chronic non-cancer pain with active substance use disorder	Recommendation against the use of opioids
4. Chronic non-cancer pain with an active psychiatric disorder, optimum non-opioid therapy, persistent pain	Stabilizing psychiatric disorder before trial of opioids
5. Chronic non-cancer pain with substance use disorder, optimum non-opioid therapy, and persistent pain	Continuing non-opioid therapy rather than a trial of opioids
6. Chronic non-cancer pain beginning long-term opioid therapy	Restrict the prescribed dose to less than 90 mg morphine equivalents daily
7. Chronic non-cancer pain beginning long-term opioid therapy	Restricting the prescribed dose to less than 50 mg morphine equivalents daily
8. Chronic non-cancer pain currently using opioids, with persistent pain and adverse effects	Rotation to other opioids
9. Chronic non-cancer pain and currently using 90 mg morphine equivalents of opioids/day	Taper opioids to the lowest effective dose and try to discontinue rather than change
10. Chronic non-cancer pain using opioids and trouble with tapering	Formal multidisciplinary program

Identifying the primary pain diagnosis allows the clinician to fully optimize evidence-supported non-opioid pharmacotherapy and non-pharmacological therapies before initiating opioids. Functioning and pain assessment should be done prior to initiating opioid therapy, along with a mental health assessment to identify potential problems that can increase the risk for opioid misuse [20].

Guidelines also emphasize physician-patient relationships that help in optimal clinical decision-making, keeping in mind every patient’s unique needs and circumstances. The recommendations should not be used as prescriptive standards of care [21]. The Pain, Enjoyment, and General Activity (PEG) scale is a tool to assess average pain (i.e., pain interference with the enjoyment of life and pain interference with general activity over the past week). This PEG can provide objective evidence to initiate a change in pain management strategies [22]. We will be further discussing spinal cord stimulation as an alternate option for chronic back pain and multidisciplinary pain rehabilitation in reducing the pain.

Spinal Cord Stimulation

A prospective, open-label clinical study of 10 kHz SCS for treating chronic back pain was undertaken at two centers in the United Kingdom and Belgium in subjects whose back pain was more than or equal to five centimeters on a 0-10 centimeters visual analog scale (VAS) and refractory to conventional treatment for more than or equal to six months [23]. VAS is a tool to help a person rate the intensity of sensations like pain. Eighty-three subjects were enrolled, including 67 with failed back surgery syndrome (FBSS). FBSS is a condition that persists or appears after spine surgery. After the implantation, the mean subject pain scores for back pain and leg pain decreased. Opioid use was reduced in 62% of the patients after the surgery with an opioid elimination rate of 38% [24]. The 10 kHz SCS had positive effects on chronic pain, including low back pain and neuropathic pain, which is often unresponsive to conventional medical treatments [11]. Retrospective reviews found back pain reductions in the range of 45-63%, as well as decreases in opioid requirements [25,26,27]. Conventional SCS treatment has been shown to be associated with a reduction in conventional opioid dose and stabilization of usage in two large retrospective studies [8,28].

Multidisciplinary Pain Rehabilitation

Opioid medications are commonly prescribed as part of a multimodal postoperative pain management strategy [6]. Post-operative clinical pain guidelines suggest opioid medications should not be continued beyond post-surgical pain or if pain persists beyond the expected period of time. There are no clear guidelines regarding the appropriate timing for tapering opioid use postoperatively. Hence, as the pain and functioning improve, the discharge education should include a plan for the reduction and discontinuation of opioids [6]. There is not much evidence on ways to best taper opioids. Dose reduction done by 20-25% every one to two days is sufficient to prevent withdrawal symptoms in patients using opioids for one to two weeks according to the recommendations by experts [29].

The Transitional Pain Service (TPS) at Toronto General Hospital (TGH) addresses chronic post-surgical pain (CPSP) in three stages. Managing patients preoperatively and postoperatively in the hospital, followed by outpatient management for six months after surgery [30]. High-risk patients for CPSP are provided comprehensive care by the multidisciplinary team of pain physicians, advanced practice nurses, psychologists, and physiotherapists after early identification [30]. The goals of TPS are to provide continuous support to preoperative and postoperative patients for pain management at increased risk of developing CPSP, support to medically complex patients, and improve functioning for an improved quality of life post-surgery [30].

The psychological context of chronic pain is explained by the connection between the patient’s emotional state and chronic pain. Chronic pain perception is affected by emotions [31]. Psychological factors have an impact on opioid use problems in patients with chronic pain, and evidence indicates that patients with psychological issues are at risk of using opioids long-term and getting involved in opioid misuse behaviors [32]. Certain studies indicate that dysfunctions in reward, appetitive, autonomic, and neurocognitive systems may be involved [32].

**Figure 2:**
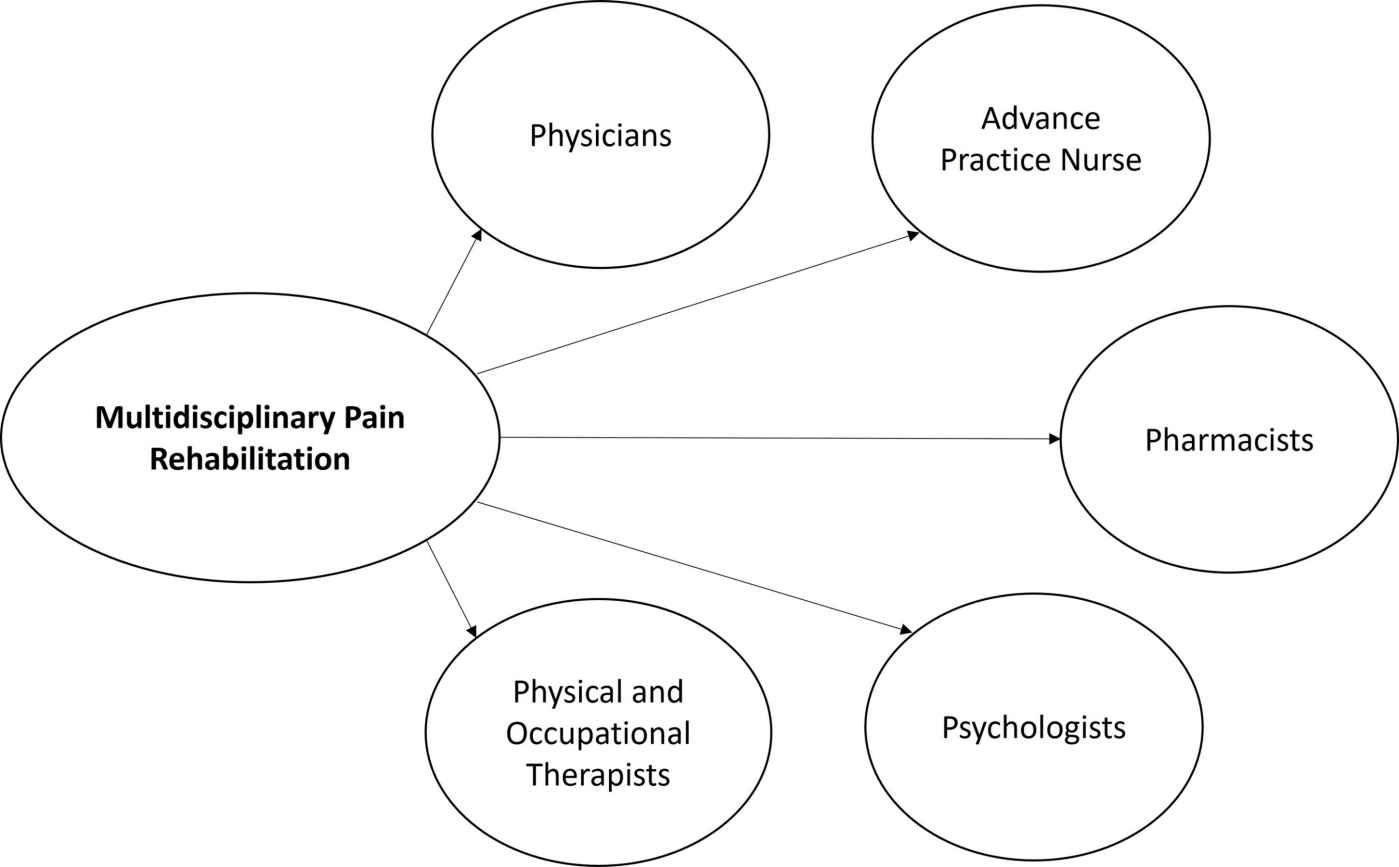
Multidisciplinary team. The image was created by the author, Sana Sultana.

Improvement in functioning is the goal of treatment through multidisciplinary treatment delivery (e.g., group-based cognitive behavioral therapy (CBT), physical therapy, and occupational therapy) that includes psychologists, physicians, physical and occupational therapists, nurses, vocational specialists, and pharmacists [33,34]. Significant improvement in pain intensity, functional disability, and sustained employment was seen with multidisciplinary pain rehabilitation [33,35]. Figure 2 shows an example of a multidisciplinary team.

An inadequate low supply of opioid medication can be related to abusive patterns or insufficient coverage of pain at a given dosage. More organized pill-counting methods can help physicians treat their patients safely and most effectively, improving the patient’s quality of life. Opioid therapy should be frequently reviewed when long-term pain control is required [12]. Prescription monitoring programs (PMPs) are linked to a decrease in opioid prescription rates [36,37].

Physical therapy for patients with musculoskeletal pain has been associated with a reduction in risk for initiating the use of opioid medication [38,39,40], but the role of physical therapy as part of a multi-modal strategy to manage co-occurring pain and opioid misuse has not been investigated. However, physical therapists can monitor patients while providing care to ensure that as a patient’s function and symptoms improve, there is an associated decrease in opioid use if this outcome is envisioned [41,42].

We need more randomized clinical trials (RCTs) to achieve effective pain control for high-risk patients in challenging situations. However, restrictive opioid consumption by using combination therapy may be considered [43]. Pain should be reassessed frequently along with functional goals, and opioids should be stopped in patients who are unresponsive [44]. Prior to starting a trial of opioid therapy, an open discussion of pain relief, functional improvements, and potential adverse effects, including the risks of an accidental overdose, death, and development of opioid use disorder, should be undertaken with a trial of non-opioid treatment first [19].

Table 3 summarises the qualitative appraisal tools used for the included studies.

**Table 3:** Qualitative appraisal tools used for the studies included. SCS: spinal cord stimulation; CNCP: chronic non-cancer pain; CPSP: chronic post-surgical pain; SANRA: Scale for the Assessment of Narrative Review; AMSTAR: Assessment of Multiple Systematic Review; OUD: opioid use disorder

Authors	Type of the study	Purpose of the study	Qualitative analysis
Nicol AL et al. [3]	Narrative review	Review the use of non-opioid analgesics for the most common non-cancer chronic pain conditions.	SANRA
Barth KS et al. [6]	Review	Review of the current literature on physician guidelines, and practitioner training.	SANRA
Page MG et al. [7]	Systematic review	Examine the relative frequency and risk factors for transitioning to long-term opioid therapy among patients who have undergone a surgical procedure or experienced trauma.	AMSTAR
Volkow N et al. [8]	Review	Overview of the contemporary problems associated with opioid management of CNCP and the related public health issues of opioid diversion, overdose, and addiction.	SANRA
Al-Kaisy A et al. [11]	Review	Summarize the current landscape of evidence in the medical literature regarding the efficacy of 10 kHz SCS to both treat pain symptoms and reduce the number of conventional opioid analgesics required by patients with chronic, non-cancer pain.	SANRA
Pergolizzi JV et al. [15]	Review	Evaluates a clinical conundrum for many practicing clinicians: these are the questions we have asked ourselves in terms of how to manage pain in patients with substance use disorder or at high risk for substance use disorder.	SANRA
Gill B et al. [16]	Systematic review	Analysis of the efficacy and practical application of pill counting during treatment of chronic pain conditions.	AMSTAR
Hooten WM. [20]	Review	Pragmatic approach to the clinical care of adults with chronic pain receiving long-term opioid therapy.	SANRA
Debono DJ et al. [30]	Review	Address the unique aspects of prescribing opioids, and provide a list of take-home points regarding the problem of chronic pain.	SANRA
Katz J et al. [32]	Review	Briefly review the main risk factors for CPSP and then describe a novel multidisciplinary pain program, the Transitional Pain Service (TPS), which has been developed and implemented over the past year.	SANRA
Hooten WM. [33]	Review	Objectives of this review were to provide a working definition of the pain matrix, which is a proposed neural network responsible for the experience of chronic pain; summarize the prevalence of commonly occurring mental health disorders in frequently encountered chronic pain conditions; and identify behavioral and pharmacological treatments with efficacy for both chronic pain and mental health disorders.	SANRA
Moride Y et al. [36]	Systematic review	Intervention to reduce or avoid opioid misuse, abuse, overdose, and diversion.	AMSTAR
Martel MO et al. [41]	Review	Highlight the predominant role played by psychological factors in the occurrence of opioid misuse and OUD in these patients.	SANRA
Magel J et al. [42]	Review	Increase physical therapists’ knowledge and skills related to managing patients taking prescription opioid medications for pain.	SANRA
Coffin PO et al. [44]	Review	Guidance for primary care clinicians on initiating, continuing, tapering opioid medication.	SANRA

Limitations

While many studies focused on opioid use and misuse, the focus on adequately addressing the pain was not extensively explored. The major limitation of our study is the inclusion of only reviews. Recommendations on current pain guidelines are also created from observational trials [6]. The CDC states the long-term safety of opioids is difficult to determine, as the RCTs of opioids for chronic pain lasted six weeks or less [11]. Further studies are needed to address adequate pain management options using non-pharmacological methods with multidisciplinary teams; this could lead to a decrease in overall opioid use and misuse.

## Conclusions

This review was conducted to determine if adequate pain management would decrease opioid use and prevent misuse. We discussed the different pain management methods in the paper, including the different pharmacotherapy options, nerve blocks, and spinal cord stimulation. The ability of SCS to reduce pain and, in turn, reduce opioid use is a positive outcome; this pain management can be used as an alternative treatment option for chronic back pain. We also talk about multidisciplinary treatment with the involvement of various departments (physician, pharmacy for pill counting, physical therapy, psychological care, etc.) in managing pain; good outcomes were observed with improvement in function. It was also observed that physical therapy reduced the initiation of opioid use. Primary care offices offering pain management should be encouraged to start therapy as a team with the involvement of psychologists and physical therapists for the best outcomes in chronic pain and to reduce opioid initiation, use, and misuse. This systematic review is important to explore the use of other treatment options for chronic pain that can reduce opioid use. Most studies were observational, which is a limitation of this paper, along with excluding the pediatric and pregnant female population. More studies of randomized control trials for a longer duration should be conducted to explore other treatment options for better pain management in patients with chronic pain.

## References

[1] (1986). Classification of chronic pain. Descriptions of chronic pain syndromes and definitions of pain terms. Prepared by the International Association for the Study of Pain, Subcommittee on Taxonomy. Pain Suppl.

[2] Smith BH, Elliott AM, Chambers WA, Smith WC, Hannaford PC, Penny K (2001). The impact of chronic pain in the community. Fam Pract.

[3] Nicol AL, Hurley RW, Benzon HT (2017). Alternatives to opioids in the pharmacologic management of chronic pain syndromes: a narrative review of randomized, controlled, and blinded clinical trials. Anesth Analg.

[4] Hylands-White N, Duarte RV, Raphael JH (2017). An overview of treatment approaches for chronic pain management. Rheumatol Int.

[5] Dowell D, Haegerich TM, Chou R (2016). CDC Guideline for Prescribing Opioids for Chronic Pain - United States, 2016. MMWR Recomm Rep.

[6] Barth KS, Guille C, McCauley J, Brady KT (2017). Targeting practitioners: a review of guidelines, training, and policy in pain management. Drug Alcohol Depend.

[7] Pagé MG, Kudrina I, Zomahoun HT (2018). Relative frequency and risk factors for long-term opioid therapy following surgery and trauma among adults: a systematic review protocol. Syst Rev.

[8] Volkow N, Benveniste H, McLellan AT (2018). Use and misuse of opioids in chronic pain. Annu Rev Med.

[9] Torrance N, Smith BH, Watson MC, Bennett MI (2007). Medication and treatment use in primary care patients with chronic pain of predominantly neuropathic origin. Fam Pract.

[10] Kawai K, Kawai AT, Wollan P, Yawn BP (2017). Adverse impacts of chronic pain on health-related quality of life, work productivity, depression and anxiety in a community-based study. Fam Pract.

[11] Al-Kaisy A, Van Buyten JP, Amirdelfan K (2020). Opioid-sparing effects of 10 kHz spinal cord stimulation: a review of clinical evidence. Ann N Y Acad Sci.

[12] Page MJ, McKenzie JE, Bossuyt PM (2021). The PRISMA 2020 statement: an updated guideline for reporting systematic reviews. BMJ.

[13] Baethge C, Goldbeck-Wood S, Mertens S (2019). SANRA-a scale for the quality assessment of narrative review articles. Res Integr Peer Rev.

[14] (2025). AMSTAR Checklist. https://amstar.ca/Amstar_Checklist.php.

[15] Pergolizzi JV, Lequang JA, Passik S, Coluzzi F (2019). Using opioid therapy for pain in clinically challenging situations: questions for clinicians. Minerva Anestesiol.

[16] Gill B, Obayashi K, Soto VB, Schatman ME, Abd-Elsayed A (2022). Pill counting as an intervention to enhance compliance and reduce adverse outcomes with analgesics prescribed for chronic pain conditions: a systematic review. Curr Pain Headache Rep.

[17] Skelly AC, Chou R, Dettori JR (2020). Noninvasive Nonpharmacological Treatment for Chronic Pain: A Systematic Review [Internet]. Agency for Healthcare Research and Quality.

[18] Von Korff M, Saunders K, Thomas Ray G (2008). De facto long-term opioid therapy for noncancer pain. Clin J Pain.

[19] (2023). The 2017 Canadian Guideline for Opioids for Chronic Non-Cancer Pain. https://app.magicapp.org/#/guideline/2849/section/34316.

[20] Hooten WM (2020). Opioid management: initiating, monitoring, and tapering. Phys Med Rehabil Clin N Am.

[21] Dowell D, Haegerich TM, Chou R (2016). CDC Guideline for prescribing opioids for chronic pain-United States, 2016. JAMA.

[22] Krebs EE, Lorenz KA, Bair MJ (2009). Development and initial validation of the PEG, a three-item scale assessing pain intensity and interference. J Gen Intern Med.

[23] Van Buyten JP, Al-Kaisy A, Smet I, Palmisani S, Smith T (2013). High-frequency spinal cord stimulation for the treatment of chronic back pain patients: results of a prospective multicenter European clinical study. Neuromodulation.

[24] Sharan AD, Riley J, Falowski S (2018). Association of opioid usage with spinal cord stimulation outcomes. Pain Med.

[25] DiBenedetto DJ, Wawrzyniak KM, Schatman ME, Kulich RJ, Finkelman M (2018). 10 kHz spinal cord stimulation: a retrospective analysis of real-world data from a community-based, interdisciplinary pain facility. J Pain Res.

[26] Stauss T, El Majdoub F, Sayed D (2019). A multicenter real-world review of 10 kHz SCS outcomes for treatment of chronic trunk and/or limb pain. Ann Clin Transl Neurol.

[27] Wilding R, Barnes S, Chincholkar M, Lalkhen A (2019). Spinal cord stimulation at 10 kHz is effective in reducing opioid consumption in patients with chronic pain. In International Neuromodulation Society 14th World Congress, May 25-30, Sydney, Australia. Spinal Cord Stimulation at 10 kHz Is Effective in Reducing Opioid Consumption in Patients With Chronic Pain.

[28] Simopoulos T, Sharma S, Wootton RJ, Orhurhu V, Aner M, Gill JS (2019). Discontinuation of chronic opiate therapy after successful stimulation is highly dependent upon the daily opioid dose. Pain Pract.

[29] Chou R, Gordon DB, de Leon-Casasola OA (2016). Management of post-operative pain: a clinical practice guideline from the American Pain Society, the American Society of Regional Anesthesia and Pain Medicine, and the American Society of Anesthesiologists' Committee on Regional Anesthesia, Executive Committee, and Administrative Council. J Pain.

[30] Debono DJ, Hoeksema LJ, Hobbs RD (2013). Caring for patients with chronic pain: pearls and pitfalls. J Am Osteopath Assoc.

[31] Institute of Medicine; Board on Health Sciences Policy; Committee on Advancing Pain Research, Care Care, and Education (2011). Relieving Pain in America: A Blueprint for Transforming Prevention, Care, Education, and Research.

[32] Katz J, Weinrib A, Fashler SR (2015). The Toronto General Hospital Transitional Pain Service: development and implementation of a multidisciplinary program to prevent chronic postsurgical pain. J Pain Res.

[33] Hooten WM (2016). Chronic pain and mental health disorders: shared neural mechanisms, epidemiology, and treatment. Mayo Clin Proc.

[34] Townsend CO, Bruce BK, Hooten WM, Rome JD (2006). The role of mental health professionals in multidisciplinary pain rehabilitation programs. J Clin Psychol.

[35] Kamper SJ, Apeldoorn AT, Chiarotto A, Smeets RJ, Ostelo RW, Guzman J, van Tulder MW (2015). Multidisciplinary biopsychosocial rehabilitation for chronic low back pain: Cochrane systematic review and meta-analysis. BMJ.

[36] Moride Y, Lemieux-Uresandi D, Castillon G, de Moura CS, Pilote L, Faure M, Bernartsky S (2019). A systematic review of interventions and programs targeting appropriate prescribing of opioids. Pain Physician.

[37] Gugelmann H, Shofer FS, Meisel ZF, Perrone J (2013). Multidisciplinary intervention decreases the use of opioid medication discharge packs from 2 urban EDs. Am J Emerg Med.

[38] Fritz JM, Kim J, Dorius J (2016). Importance of the type of provider seen to begin health care for a new episode low back pain: associations with future utilization and costs. J Eval Clin Pract.

[39] Kazis LE, Ameli O, Rothendler J (2019). Observational retrospective study of the association of initial healthcare provider for new-onset low back pain with early and long-term opioid use. BMJ Open.

[40] Frogner BK, Harwood K, Andrilla CH, Schwartz M, Pines JM (2018). Physical therapy as the first point of care to treat low back pain: an instrumental variables approach to estimate impact on opioid prescription, health care utilization, and costs. Health Serv Res.

[41] Martel MO, Bruneau A, Edwards RR (2021). Mind-body approaches targeting the psychological aspects of opioid use problems in patients with chronic pain: evidence and opportunities. Transl Res.

[42] Magel J, Kietrys D, Kruger ES, Fritz JM, Gordon AJ (2021). Physical therapists should play a greater role in managing patients with opioid use and opioid misuse. Subst Abus.

[43] Lowry R (2018). Using drug monitoring programs to optimize pain management for elective surgery patients. JAAPA.

[44] Coffin PO, Martinez RS, Wylie B, Ryder B (2022). Primary care management of long-term opioid therapy. Ann Med.

